# Molecular and Functional Characterization of a Wheat B2 Protein Imparting Adverse Temperature Tolerance and Influencing Plant Growth

**DOI:** 10.3389/fpls.2016.00642

**Published:** 2016-05-10

**Authors:** Akanksha Singh, Paramjit Khurana

**Affiliations:** Department of Plant Molecular Biology, University of DelhiNew Delhi, India

**Keywords:** ABA, *Arabidopsis*, B2 protein, high temperature stress, wheat

## Abstract

Genomic attempts were undertaken to elucidate the plant developmental responses to heat stress, and to characterize the roles of B2 protein in mediating those responses. A wheat expressed sequence tag for B2 protein was identified which was cloned and characterized to assess its functional relevance causing plant growth and development during stress adaptation. Here, we show that wheat B2 protein is highly expressed in root and shoot tissues as well as in developing seed tissues under high temperature stress conditions. Morphological studies of transgenic *Arabidopsis* overexpressing gene encoding wheat B2 protein and Δ*b2* mutant plants were studied at major developmental stages. The stunted growth phenotype of mutant plants, together with hypocotyl and root elongation analysis of transgenic plants showed that B2 protein exhibits a crucial role in plant growth and development. Additional physiological analyses highlights the role of B2 protein in increased tolerance to heat and cold stresses by maintaining high chlorophyll content, strong activity of photosystem II and less membrane damage of overexpression transgenics as compared with the wild-type. Furthermore, the constitutive overexpression of *TaB2* in *Arabidopsis* resulted in ABA hypersensitivity. Taken together, these studies suggest a novel perspectives of B2 protein in plant development and in mediating the thermal stress tolerance.

## Introduction

Plants are exposed to a plethora of stress conditions like heat, cold, salinity, drought, osmotic, and oxidative stress. In fact abiotic stress is the main reason for low yield of crops worldwide, limiting growth and productivity (Mickelbart et al., 2015). Among all these stresses, thermal stress tolerance appears to be the major challenge for plants (Kotak et al., 2007). Temperature variations such as high temperatures, cold, or freezing has strong effects on the architecture of plants (Miura and Furumoto, 2013) and reduction in yield of crops (Boyer, 1982). Wheat is a temperate crop therefore sensitive to extreme temperatures during mid-anthesis stages affecting seed fertilization and seed set ultimately lowering its yield (Ferris et al., 1998). Since complete genome sequence of wheat has become available only recently, there were not enough studies related to high temperature stress, as compared to those in *Arabidopsis* and rice. This could be attributed to the complex genome and a relatively longer life cycle of wheat (Chauhan et al., 2013). With the aim to unfold the complexity of temperature stress response in wheat, putative heat stress related genes were identified from subtractive heat stress library of wheat (Chauhan et al., 2011).

The putative B2 protein, a novel gene was found to participate in environmental stress conditions and plant development (Tenhaken et al., 2005). B2 protein was initially identified in carrot (*Daucus carota*) where it was found to be strongly induced during the developmental shift from undifferentiated cell cultures to cultures displaying somatic embryogenesis (Schrader et al., 1997). B2 protein comprises of a novel plant development specific and cell death domain (DCD) whose amino acids are found to be well conserved among the plant kingdom members and are not present in bacteria, fungi and animals. DCD-domain has proteins clustered into four different subgroups, depending on the location of the domain within the protein (Tenhaken et al., 2005). In general, many heat shock proteins (Chang et al., 2007), rubisco activase (Ristic et al., 2009), lipid transfer protein (Wang et al., 2014) impart heat stress tolerance in crop plants, however, putative B2 protein could be an advantage over the known proteins because its expression pattern was found in the developing seed tissue of wheat (Chauhan et al., 2011) which is responsible for the optimization of late embryogenesis.

In the present study we cloned and characterized a putative B2 protein from *Triticum aestivum*, from the expressed sequence tag (EST) collection of a heat stress subtractive cDNA library of developing seed (Chauhan et al., 2011). Despite the growing importance of EST resources in wheat, their molecular and functional relevance under abiotic stress tolerance and in plant development needs to be elucidated. Here, we report the sequence analysis of *TaB2* and expression profile in varied tissues and during major developmental stages for *TaB2*. The overexpression (OE) of *TaB2* in *Arabidopsis* enhanced the hypocotyl growth, increased root length, plant height, and generated healthy rosettes. To investigate its role in response to high and low temperature stress, morphological and physiological experiments were performed in *TaB2*-OE, WT, *b2* mutant (Δ*b2*) and mutant complemented (*C*Δ*b2*) seedlings. Furthermore, to gain new insights in ABA stress signaling, germination and seedling growth response of *TaB2* overexpression transgenic lines in *Arabidopsis* were analyzed. Hence, our results show that putative plant B2 proteins are possibly involved in stress responses and play an elemental role in plant growth and developmental.

## Materials and Methods

### Plant Material, Growth Conditions, and Stress Treatments

For expression analysis of B2 protein coding gene in wheat, *T. aestivum* cv. PBW343 seeds were surface sterilized, placed in the culture room maintained at 22 ± 1°C with 16:8 h light and dark phase with a light intensity of 100–125 μmol m^-2^s^-1^. For gene expression analysis, 12-day-old seedlings were given heat stress at 37°C for 2 h and then at 42°C for another 2 h maintained in a growth chamber. In addition, seedlings were incubated in 150 mM NaCl solution for simulating salt stress, 2% mannitol for drought stress and at 4°C for cold stress for 24 h. For hormone treatments, seedlings were immersed in abscisic acid (ABA; 10 μM, Sigma, USA), brassinosteroid (BR; 1 μM epibrassinolide, Sigma), salicylic acid (SA; 100 μM) and CaCl_2_ (10 mM) solutions for 4 h (Khurana et al., 2012). All samples were frozen in liquid nitrogen and stored at -80°C until RNA isolation. *Arabidopsis thaliana* ecotype Columbia was used as wild-type for the generation of transgenic plants and for gene expression analysis. Plants were grown in Petriplates containing half strength of MS medium and in pots containing vermiculite, with a 16:8 h light and dark phase at 22 ± 1°C under 100–125 μmol m^-2^s^-1^ photoperiod.

### Isolation and Molecular Cloning of *TaB2* by 5′ and 3′ RACE

A partial sequence of the putative *TaB2* cDNA obtained from the developing seed subtractive library of heat-stressed tissue (Chauhan et al., 2011) was used as the starting sequence to obtain the full length cDNA. In order to amplify the complete gene, 5′ and 3′ RACE using gene- specific primers (GSP) and nested primers (NGSP) were designed using Gene Runner software^1^ from its 5′ and 3′ end. Primers used for 5′ and 3′ RACE PCR reactions are listed in Supplementary Table S1 and protocol was followed as described in the manual BD SMART RACE kit (Clontech, USA). The amplified RACE- PCR products were cloned into pDRIVE cloning vector and sequenced by ABI PRISM Big Dye Terminator version 3.1 cycle sequencing kit (Applied Biosystems). After the alignment of RACE products, a full TaB2 encoding gene sequence was amplified using RNA from heat stressed developing seed tissue by SuperScript III one- step RT-PCR system for long templates (Invitrogen, USA) using gene specific primers (Supplementary Table S1), cloned in pDRIVE (Qiagen) vector and sequenced by an ABI PRISM3700 DNA sequencer (USA).

### Sequence Analysis and Phylogenetic Relationship

To identify the wheat B2 protein, cloned in the present study, the nucleotide and protein sequence were analyzed using Gene Runner Program^2^ (version 3.04). Domain search program SMART^3^ was used to infer the domain structure of putative protein sequence. The nucleotide and deduced amino acid sequence searched for their homology from *Hordeum vulgare* (BAJ90431), *Brachypodium distachyon* (XP_003568175), *A. thaliana* (NP_189345), *Solanum lycopersicum* (XP_004233294), *Vitis vinifera* (XP_002269664.1), *Cucumis sativus* (XP_004145666), *Populus trichocarpa* (XP_006373229.1), *Glycine max* (XP_006584575.1), *Oryza brachyantha* (XP_006654598), *Zea mays* (NP_001148917.1), *Ricinus communis* (XP_002518847.1), *Fragaria vesca* (XP_004303971.1), were obtained from GenBank^4^. Phylogenetic tree of TaB2 was constructed using the NJ (neighbor-joining) method in MEGA (version 6) software program.

### Subcellular Localization of TaB2

The complete open reading frame (ORF) of TaB2 protein was fused to green fluorescent protein (GFP) reporter gene in frame under the control of the cauliflower mosaic virus 35S promoter (CaMV 35S) in pSITE-2CA vector. About 2 μg of the plasmid construct was used to coat gold particles. Inner epidermal peels of white onion were placed inside-up on MS medium. Onion peels were bombarded by using PDS-1000/He system (Bio-Rad, Canada) at 1,100 p.s.i. with DNA coated gold particles and 6 cm of target distance using 1.0 μm of gold micro-carriers. After bombardment, the Petriplates were sealed with parafilm and incubated overnight at 28°C before observation. The onion epidermal cells were observed by using confocal microscope (Leica^®^ TCS SP5II).

### RNA Extraction and Quantitative Real-Time PCR

RNA from wheat seedlings and different *Arabidopsis* plants was isolated by RNeasy Plant Mini Kit (Qiagen, Germany) according to the manufacturer’s protocol followed by on-column DNase-I treatment for removal of genomic DNA contamination. Total RNA from developing wheat seeds of different stages viz., 7, 15, and 20 DAA (days after anthesis) and mature seeds were isolated as per the method described by Singh et al. (2003). First strand cDNA was synthesized with 2 μg of total RNA using the High Capacity cDNA Archive kit (Applied Biosystems, USA) and 200 nM of each primer mixed with SYBR Green PCR Master Mix (Applied Biosystems) for real-time PCR reactions, using the ABI Prism 7000 Sequence Detection System and Software (PE Applied Biosystems) according to the manufacturer’s instruction. Primers for real time PCR analysis were designed using primer design software ‘Primer Express version 2.0’ (Applied Biosystems^®^, USA) and each pair was confirmed by BLAST program in NCBI and TAIR database. The specificity of the PCR reactions was verified by the melting curve analysis. Primers used for Real- time PCR were mentioned in Supplementary Table S1. The relative mRNA levels in different RNA samples were normalized with respect to internal control gene, *Actin*. The Ct (threshold cycles) values were averaged for two biological replicates and three technical replicates and plotted using the relative mRNA value in each case.

### Construction and Generation of Transgenic *Arabidopsis* Plants

For over-expression studies, a 1.396 kb fragment (cDNA) containing the wheat B2 protein encoding gene ORF along with 5′ and 3′ UTR, was PCR amplified from pDRIVE:*TaB2* plasmid with TOPO-*TaB2*-FP and *TaB2*-RP primers (Supplementary Table S1). The amplified product was inserted into pENTR/D-TOPO vector (Invitrogen Life technologies, Carlsbad, CA, USA) for construction of entry clone and sequenced. The selective positive recombinant was then mobilized into the destination vector pMDC32 by LR-clonase reaction (Gateway LR Clonase II, Invitrogen Life Technologies, Carlsbad, CA, USA) to construct pMDC32:*TaB2* vector. To generate transgenic overexpression plants *TaB2*: pMDC32 was introduced into *Agrobacterium tumefaciens* strain *AGL1* by freeze thaw method and then the selective recombinant transformed into *A. thaliana* ecotype Col-0, wild type by floral dip method (Clough and Bent, 1998). T-DNA mutant plants for *AtB2*, SALK_044868 and SALK_041306 obtained from the *Arabidopsis* Biological Resource Center (ABRC) were used for further analyses. These Δ*b2* seeds were multiplied, verified and screened for homozygous lines through PCR. Genomic DNA was isolated from approximately five individual plants per mutant line. Two paired PCR reactions used containing left border primer (LB) of the T-DNA insertion specific and right gene specific primer (RP) in one reaction whereas other reaction used left and right gene specific primer (LP and RP) designed by Signal isect toolbox^5^ (Supplementary Table S1). Homozygous lines for the T-DNA insertion was verified when PCR reaction yielded only one product. These confirmed homozygous lines were then transformed by floral dip method to obtain its complemented lines. For analyses of different transgenic lines under various abiotic stresses, T4 homozygous seeds were used and the results presented represents at least three independent experiments. The data presented represents at least 10 replicates and values represented are average means of these experiments with standard error bars.

### Hypocotyl Elongation Growth Assay

For hypocotyl elongation growth assay, seeds were germinated on MS (Murashige and Skoog, 1962) medium and placed in dark conditions at 22°C for 2.5 days. Gradient heat stress was given to the seeds initial at 38°C for 90 min, then allowed to recover at room temperature for 120 min and again subjected to heat stress at 42°C for 180 min. Seedlings were then observed for their growth after an additional 2.5 days in dark. Hypocotyl length of 20 seedlings each from wild type, mutant, transgenic lines and its complemented lines were measured and averaged. Graph was plotted as percentage increase in hypocotyl elongation. Hypocotyls were pulled straight during measurement using forceps and photographed.

### Root Growth Inhibition Assay

To assess the root growth under high and low temperature stress treatments, wild-type, mutant, transgenic and complement seeds were grown on MS medium (supplemented with 2% sucrose and 0.8% agar) in plates kept vertically for 4 days at 22°C, under a 16 h light and 8 h dark regimen, in a culture room. 4-day old seedlings were given heat stress at 42°C for 120 min and then the plants were allowed to grow for 5 days. Also, cold stress for 24 h at 4°C was given to the 4-day old seedlings. The position of root tip was marked before heat stress and cold stress treatment. After 5 days of growth, root elongation was quantified. Ten seedlings from each transgenic line, mutant, complement, and wild-type plants were measured for root elongation and plotted as percentage increase in root elongation.

### Physiological and Biochemical Analysis of Transgenics

#### Chlorophyll Fluorescence Measurements

Photosystem II activity was measured using a pulse amplitude modulation fluorometer (Junior PAM-210, H. Waltz, Germany) at room temperature. The Perspex light guide that delivers the measuring and saturating light was held in close contact with the upper surface of the leaves which were dark adapted for 20 min before measuring the induction of fluorescence (Checker and Khurana, 2013). The measuring beam was used to induce the minimum fluorescence (*F*_0_). Maximum photosynthetic efficiency (*F*v/*F*m) which is proportional to potential maximal quantum yield of PSII (Krause and Weis, 1991) was recorded under control, high and low temperature stress treated rosette leaves of atleast ten plants viz. wild-type, mutant, overexpression, and complemented lines.

#### Measurement of Total Chlorophyll Content

Chlorophyll content was estimated by the non-maceration method according to Hiscox and Israelstam (1979). Leaf samples (0.05 g) from control and stress treated plants were incubated in 5 mL of DMSO at 65°C for 4 h in dark and absorbance recorded at 645 and 633 nm in UV- Vis spectrophotometer (Agilent Cary 60). Chlorophyll content was calculated according to the formula of Arnon (1949).

#### Membrane Stability Index (MSI)

Membrane stability index was estimated by recording the electrical conductivity according to the Sairam et al. (2002) protocol. Leaf tissue (0.1 g) was taken in double distilled water and initial conductivity (C1) measured after keeping control and stressed samples at 30°C for 30 min. The samples were then autoclaved for 15 min and set to cool down and electric conductivity (C2) was measured by conductivity meter (Eutech instrument, Singapore).

$$\mathrm{MSI}\;\mathrm{was}\;\mathrm{calculated}\;\mathrm{using}\;\mathrm{formula}:\mathrm{MSI}\;=\;{\left\{1-(C1/C2)\right\}}^{*}100$$

#### Lipid Peroxidation Assay

The thiobarbituric acid (TBA) test, which determines MDA as an end product, was used to analyze lipid peroxidation (Heath and Packer, 1968; Hodges et al., 1999). Briefly, 0.2 g plant material was homogenized in 4 mL of 0.1% (w/v) trichloroacetic acid (TCA) solution kept on ice. The suspension was rinsed into a centrifuge tube with an additional 1 mL of TCA. The homogenate was centrifuged at 10,000 *g* for 5 min, and the supernatant collected. 1 mL of 20% (w/v) TCA containing 0.5% (w/v) TBA was added to a 0.5 mL aliquot of the supernatant. The mixture was kept in a boiling water bath for 30 min and then quickly cooled in an ice bath. After centrifugation at 10,000 *g* for 10 min, the absorbance of the supernatant was measured at 532 and 600 nm. The MDA concentration was calculated according to the Heath and Packer (1968).

$$\mathrm{MDA}\;\mathrm{equivalents}(\mathrm{nmol}.{\mathrm{ml}}^{-}{}^{1})\;=\;[(A532-A600)/155000]10^{6}$$

#### *In Situ* Detection of Leaf Superoxide ($O_{2}^{•–}$)

To detect the presence of superoxide ($O_{2}^{•–}$) levels in wild-type, mutant, transgenic and complemented *Arabidopsis* plants, nitro blue tetrazolium (NBT) stain was used. The detached leaves of 3-week old *Arabidopsis* plants grown under unstressed culture conditions and 2 h high temperature stress at 40°C conditions were stained in 0.1% NBT solution dissolved in 10 mM potassium phosphate buffer (pH 6.4) for detection of $O_{2}^{•–}$ incubated at room temperature for 4 h in dark. After incubation for 4 h, the stained leaves were kept in destaining solution containing 3:1:1 ethanol: acetic acid: glycerol and boiled at 100°C (Jambunathan, 2010). Superoxide ions react with NBT and detected with blue stain. The stained leaves photographed with the help of stereo microscope Leica DFC 290 HD.

## Results

### Cloning and Sequence Analysis of *TaB2*

**FIGURE 1:**
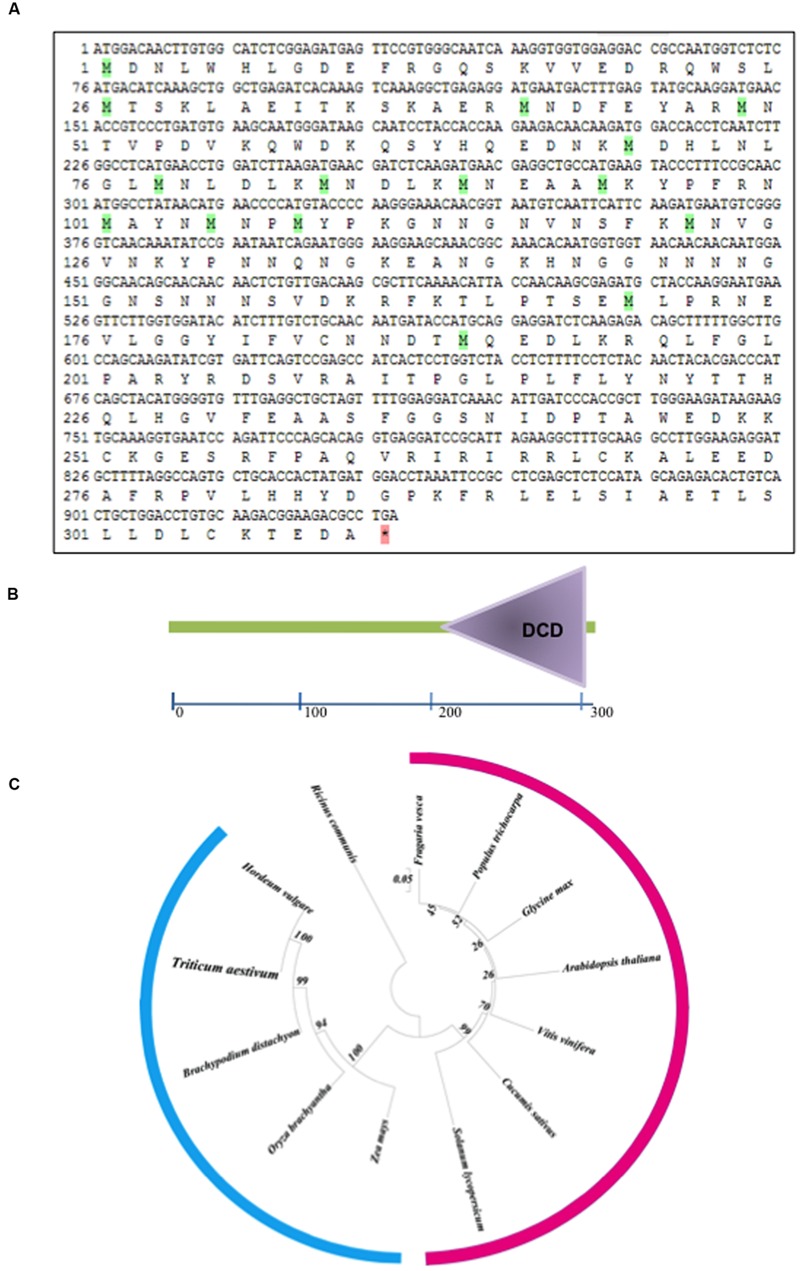
**Structural organization of *TaB2.* (A)** Open reading frame (ORF) and protein sequence of *TaB2.* **(B)** Domain structure of TaB2 protein highlighting the position of development and cell death domain (DCD) present in the protein structure. **(C)** Phylogenetic tree of TaB2 constructed by neighbor-joining (NJ) method using MEGA (version 6) software with its homologs across various monocot (blue color) and dicot (pink color) plant species. Bootstrap values out of 100 replicate data sets have been displayed at the branch nodes.

Through northern analysis, the expression of putative B2 protein encoding gene transcript was found to be highly expressed at 42°C (Chauhan et al., 2011). The full length cDNA of 1,396 bp (**Figure 1A**) was obtained by comparison of the sequence of the initial clone and the RACE products harboring 933 bp ORF with 128 bp of 5′ UTR and 335 bp of 3′ UTR encoding 310 deduced amino acid residues with a calculated molecular mass of 35.62 kDa and a predicted isoelectric point of 8.35. The protein sequence of TaB2 contains development and cell DCD from position 175 to 307 amino acid residues analyzed by SMART (Smart Modular Architecture Research Tool) database (**Figure 1B**). This DCD domain is found particularly in plant proteins involved in development and cell death. A neighbor-joining (NJ) phylogenetic tree was constructed in Mega (version 6) software using ORF sequences of putative B2 protein (**Figure 1C**). It was evident from the tree that the putative *TaB2* cloned in the present study formed a tight group with other monocots and closely related to *H. vulgare* whereas dicot species sharing same DCD domain clustered altogether in a different clade. Multiple sequence alignment analysis using deduced amino acid from BLASTp in NCBI database revealed that putative B2 protein showed high similarity of 99% with *H. vulgare* predicted protein which is not yet annotated (Supplementary Figure S1). A high sequence similarity was also found with other monocots like *B. distachyon, Z. mays, O. brachyantha*, however, when compared with dicots, *A. thaliana*, B2 protein showed only 59% similarity with *T. aestivum* B2 protein.

### Subcellular Localization of TaB2

**FIGURE 2:**
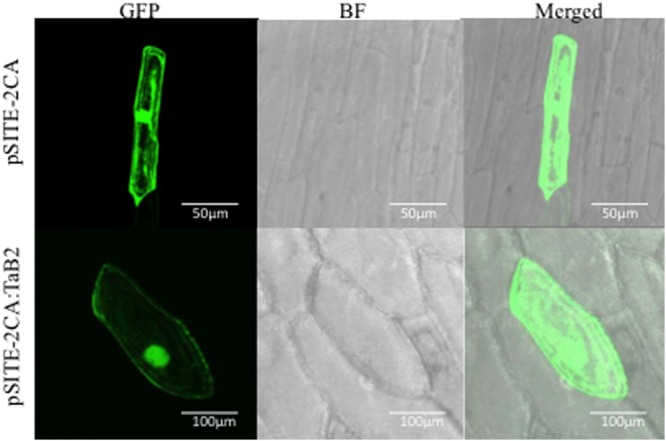
**Sub-cellular localization of TaB2.** The chimeric protein (pSITE-2CA:TaB2) was localized to the nucleus as well as in the cytoplasm while in control (pSITE-2CA), GFP localized throughout the cell.

The Confocal microscopy of transformed onion epidermal cells revealed thatTaB2-GFP fusion protein localized in nucleus and cytoplasm. As expected empty vector GFP protein showed fluorescence distributed throughout the cell (**Figure 2**).

### Expression Analysis of *TaB2* at Major Developmental Stages and in Response to Abiotic Stress

**FIGURE 3:**
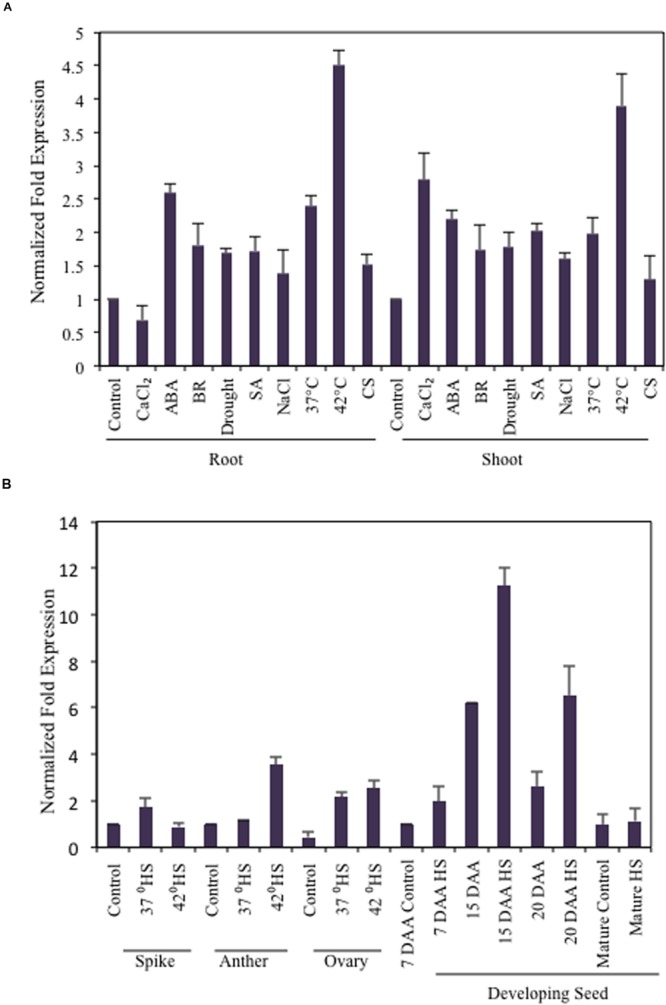
**Expression analysis of *TaB2.*** Quantitative RT-PCR analysis in different abiotic stresses and growth effectors **(A)** and at major developmental stages under non- stressed and heat stress conditions. **(B)** Wheat *Actin* used as an internal control and the experiment was repeated three times.

The expression profile of *TaB2* was analyzed in 35 different combination of tissues under various abiotic stress treatments along with some growth effectors at major developmental stages of wheat (**Figures 3A,B**). Interestingly, in vegetative tissues *TaB2* showed higher expression during high temperature stress at 42°C in both root and shoot. It also displayed upregulation to exogenous ABA treatment suggesting that *TaB2* may be regulated via an ABA-mediated signaling pathway. However, comparatively low level of expression were observed in seedling tissues when induced by BR, SA, CaCl_2_, NaCl, drought, and cold. Differential expression analysis was also observed at major developmental stages of wheat where *TaB2* displayed moderate expression (fold change ≥3) specifically in high temperature stressed (42°C) anther tissue as compared to the spike and ovary. However, *TaB2* is highly inducible by high temperatures in developing seeds especially in 15 DAA seed stage followed by 20 DAA.

### Overexpression of *TaB2* and Screening of Mutant SALK Lines in *Arabidopsis*

**FIGURE 4:**
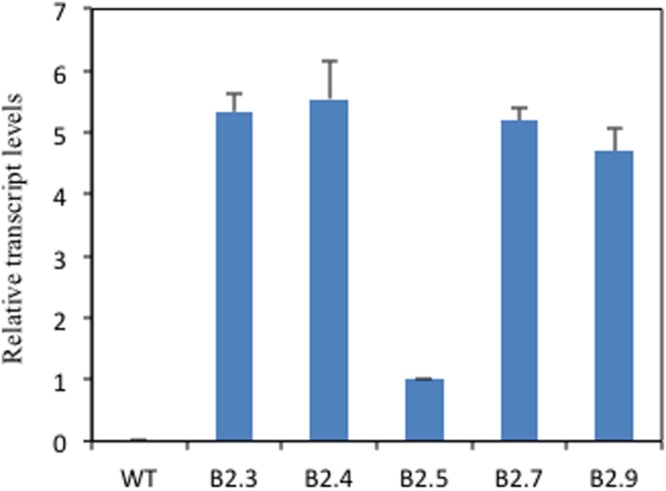
**Overcxprcssion of *TaB2* in transgenic *Arabidopsis* plants.** Real- lime PCR analysis of WT and *TaB2* transgenic *Arabidopsis* plants, showing the expression in five homozygous transgenic lines (B2.3, B2.4, B2.5, B2.7, and B2.9) with no detectable expression in WT relative to B2.5 line indicating the lowest expression of the transgene. *Actin* gene was used as an internal control. Data are mean ± SE from two biological and three technical replicates.

To elucidate the role of putative TaB2 protein in plant development and abiotic stress, transgenic *Arabidopsis* plants overexpressing *TaB2* under the control of CaMV 35S promoter were raised. The transgenic nature of putative transformants of *TaB2* in *Arabidopsis* was confirmed by PCR (Supplementary Figure S2). The transcript level of transgenic lines B2.3, B2.4, and B2.7 showed higher expression than B2.5 and B2.9 whereas no detectable expression monitored in wild-type (**Figure 4**). Therefore, on the basis of transcript level three transgenic lines (B2.3, B2.4, and B2.7) of *Arabidopsis* were selected for further morphological and phenotypic analysis in T4 generation. Also, we selected T-DNA insertion mutant SALK lines of *Arabidopsis* putative B2 protein coding gene from TAIR database SALK_044868 and SALK_041306. As a result of PCR, from SALK_044868, mutant line Δ*M1.12* and SALK_041306 mutant line Δ*M2.6* was selected for further transgenic analysis (Supplementary Figure S3).

### Constitutive Expression of *TaB2* Rescues Δ*b2* Phenotype in *Arabidopsis*

**FIGURE 5:**
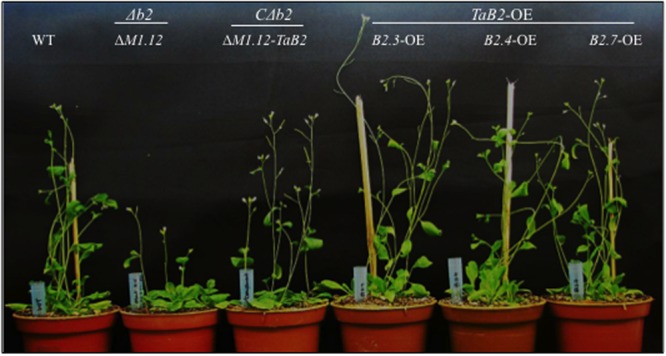
**Morphometric analysis of WT, mutant, complemented *(CΔb2)* and overexpression (OE) lines of *TaB2.*** Phcnotype of WT, Δ*M1.12*,Δ*M1.12: TaB2* and *TaB2-* OE transgenic lines of 3 weeks old *Arabidopsis* plants.

To compare the phenotypic traits among WT, Δ*b2, TaB2-OE* and *C*Δ*b2* plants, we examined the morphometric details. The Δ*b2* plant viz. Δ*M1.12* showed stunted growth as compared to the *C*Δ*b2* plant (Δ*M1.12*:*TaB2*) which were found to be phenotypically identical to wild-type. However, overexpression transgenic plants of *TaB2* showed faster germination and exhibited more developed inflorescence and better silique formation (**Figure 5**). The mutant Δ*M1.12* plants showed short plant height, reduced rosette diameter, lesser rosette leaf number, reduced leaf length and width, reduced and smaller number of siliques per plant (**Table 1**). The stunted growth of Δ*b2* plant was rescued by *C*Δ*b2* plant, which implies the importance of *TaB2* in development. The *TaB2-OE* plants were characterized by increased plant height, rosette diameter, rosette leaf number, enlarged leaf length, leaf width, silique length, and more number of siliques per plant with significant difference as compared to the WT and *C*Δ*b2* plants, respectively.

**Table 1:** Morphometric comparison among WT and *TaB2 Arabidopsis* transgenics.

Morphological characteristics	WT- Col0	Δ*b2*	*C*Δ*b2*	*B2.3*-OE	*B2.4*-OE	*B2.7*-OE
Plant height (cm)^a^	15.2 ± 0.21	9 ± 0.63	16.2 ± 0.37	21.8 ± 0.35	23.2 ± 0.37	23.6 ± 0.25
Rosette diameter (cm)^b^	1.8 ± 0.02	1.5 ± 0.04	1.9 ± 0.06	2.02 ± 0.01	2.2 ± 0.06	1.9 ± 0.03
Rosette leaf no.^b^	8.8 ± 0.05	6.2 ± 0.64	8.6 ± 0.06	10.2 ± 0.13	11.3 ± 0.25	10.8 ± 0.29
Leaf length (cm)^c^	1.5 ± 0.12	0.8 ± 0.07	1.2 ± 0.12	1.92 ± 0.11	1.8 ± 0.13	1.9 ± 0.12
Leaf width (cm)^c^	0.9 ± 0.08	0.9 ± 0.52	0.9 ± 0.42	1.36 ± 0.07	1.2 ± 0.03	1.3 ± 0.09
Silique no./plant	10 ± 0.61	8.2 ± 0.41	12.4 ± 0.37	17.6 ± 0.04	16.8 ± 0.09	13 ± 0.07
Silique length (cm)	1.2 ± 0.08	0.9 ± 0.05	1.6 ± 0.08	1.98 ± 0.10	1.9 ± 0.09	2.0 ± 0.07

*^a^Maximum height of 3-week-old plant, ^b^At the time of bolting, ^c^of the largest leaf at the time of bolting.*

### Expression Profile of *TaB2* Overexpression in *Arabidopsis*

**FIGURE 6:**
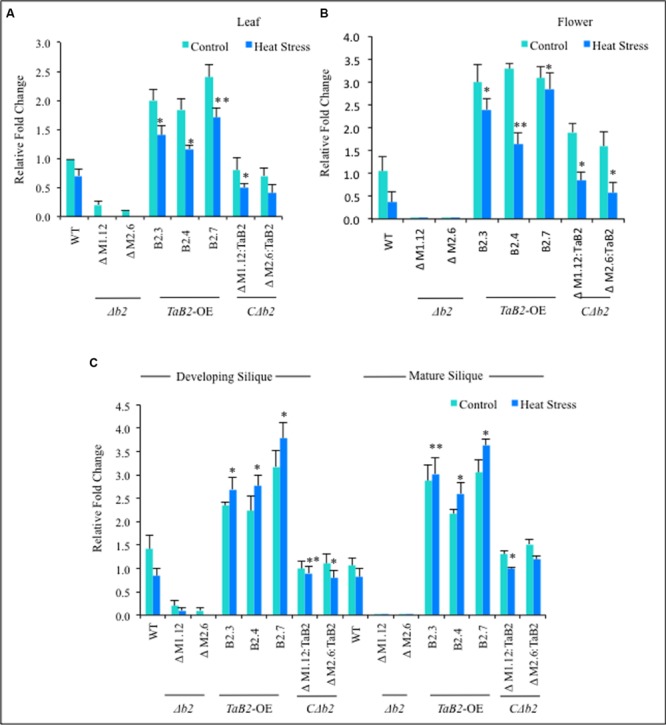
**Quantitative RT-PCR was done in different tissues viz. (A)** leaf, **(B)** Flower, **(C)** developing and mature siliquc of 8-week old *Arabidopsis* plants of WT, Δ*b2, TaB2-OE* and *C*Δ*b2* under control and heat stress conditions. Data represents from three independent replicates and are mean ± SD. Asterisks marked above bars indicate statistically significant differences (*^∗^P* ≤ 0.05, ^∗∗^*P* ≤ 0.01).

The expression behavior of WT, *Δb2, C*Δ*b2*, and *TaB2*-OE encoding gene transgenics, was undertaken by real time PCR in different tissues viz. leaf, flower, developing and mature siliques under control and high temperature stressed conditions. We observed that in leaf and flower tissues the relative fold-change of expression was not significantly increased at high temperature as compared to the non-stressed *Arabidopsis* plants (**Figures 6A,B**), however, the expression of *TaB2* transgenic line was upregulated in developing and mature siliques under high temperature stress, as compared to the control conditions (**Figure 6C**). Further in Δ*b2* lines, negligible expression was observed in all the *Arabidopsis* tissues under non-stressed and heat stress conditions whereas no significant difference was observed in the expression level of *C*Δ*b2* lines with respect to the WT. Thus the above analysis indicates that *TaB2* functions during both seed development and high temperature stress response.

### Enhanced Hypocotyl Elongation of *TaB2* Transgenic *Arabidopsis* Plants

**FIGURE 7:**
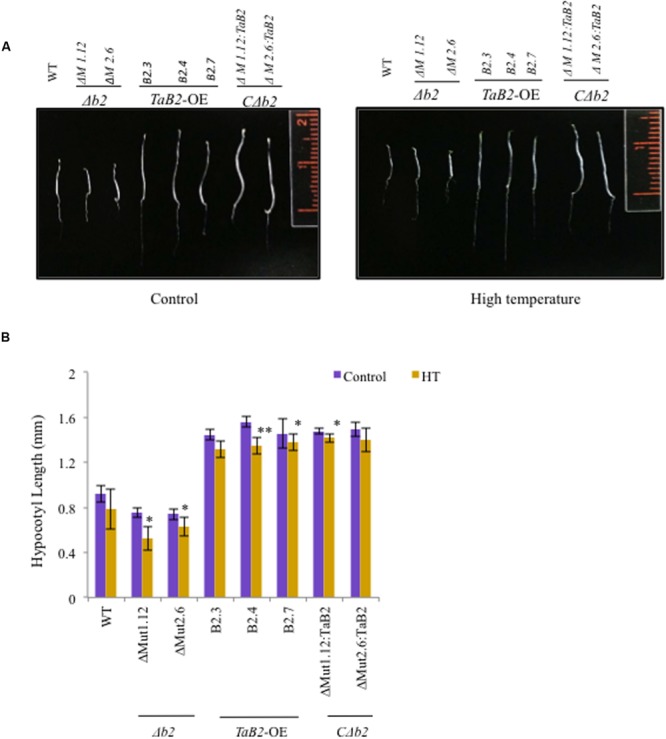
**Hypocotyl elongation assay of 5-day-old *Arabidopsis W*T, Δ*b2, TaB2* -OE, and *C*Δ*b2* seedlings grown under control and high temperature (HT) stress conditions. (A)** Phenotype of the hypocotyl of different *Arabidopsis* seedlings. **(B)** Histogram representing the hypocotyl length of WT, Δ*b2, TaB2* -OE, and *C*Δ*b2* seedlings. Graph plotted taking ±SD of 20 seedlings for each *Arabidopsis* lines. Asterisks marked above bars indicate statistically significant differences (^∗^*P* ≤ 0.05, ^∗∗^ *P* ≤ 0.01).

For hypocotyl elongation assay, the T4 generation *TaB2*-OE seedlings were tested for acquired thermotolerance and high temperature stress. The 5-day-old *TaB2* transgenic and *C*Δ*b2* seedlings displayed significantly longer hypocotyl when compared with the WT and Δ*b2* seedlings grown under normal temperature conditions (**Figure 7A**). However, under high temperature stress conditions the hypocotyl length decreases in WT, transgenic, Δ*b2* and *C*Δ*b2* lines. Both mutant lines Δ*M1.12* and Δ*M2.6* failed to show hypocotyl elongation under normal and high temperatures while *C*Δ*b2* lines rescued this phenotype. The hypocotyl length was then measured, plotted and photographed (**Figure 7B**).

### Overexpression of *TaB2* Enhanced Tolerance to Altered Temperature Stress

**FIGURE 8:**
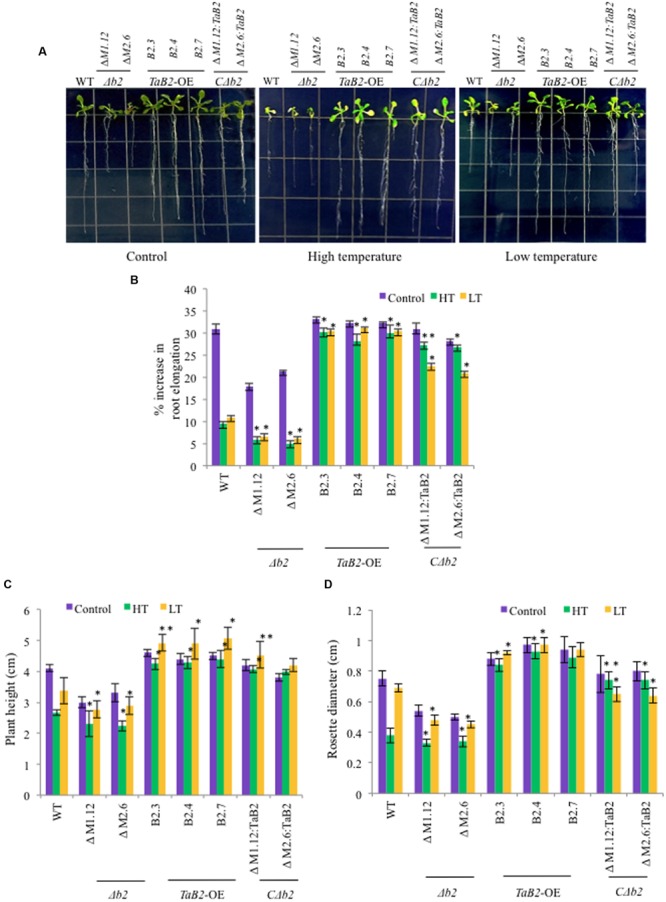
**Phenotypic analysis of *TaB2*-OE transgenics in *Arabidopsis* under high (HT) and low (LT) temperature stress. (A)** Root length phenotype of WT, Δ*b2, TaB2* -OE, and *C*Δ*b2* lines grown on MS medium 7 days after high (42°C) and low (4°C) temperature stress was given. **(B)** Effect of adverse temperature stress on root length expressed as percentage increase, **(C)** plant height and **(D)** rosette diameter. Values plotted arc mean for 10 seedlings each and data represents are mean ± SD from three independent replicates. Asterisks marked above bars indicate statistically significant differences (^∗^*P* ≤ 0.05, ^∗∗^*P* ≤ 0.01).

We checked the response of overexpression transgenic lines, Δ*b2, C*Δ*b2*, and WT plants under high and low temperature stress conditions. In root growth assays, the transgenic and *C*Δ*b2* lines had much elongated roots as compared to the WT and Δ*b2* seedlings at high and low temperatures while there is no significant difference observed in root length under control conditions except in the Δ*b2* plants where the extent of retardation in root length was higher (**Figures 8A,B**). We observed increased plant height and larger rosette diameter of transgenic as compared to the Δ*b2* and WT under high and low temperature stress conditions whereas under non-stressed condition no significant difference was found except in the Δ*b2* seedlings where it decreases markedly (**Figures 8C,D**). Also, the rosette leaves in *Arabidopsis* plants under high temperature stress remained green in transgenic lines as compared to the WT, however, under low temperature stress conditions no phenotypic difference displayed relative to the rosettes between transgenics and WT.

**FIGURE 9:**
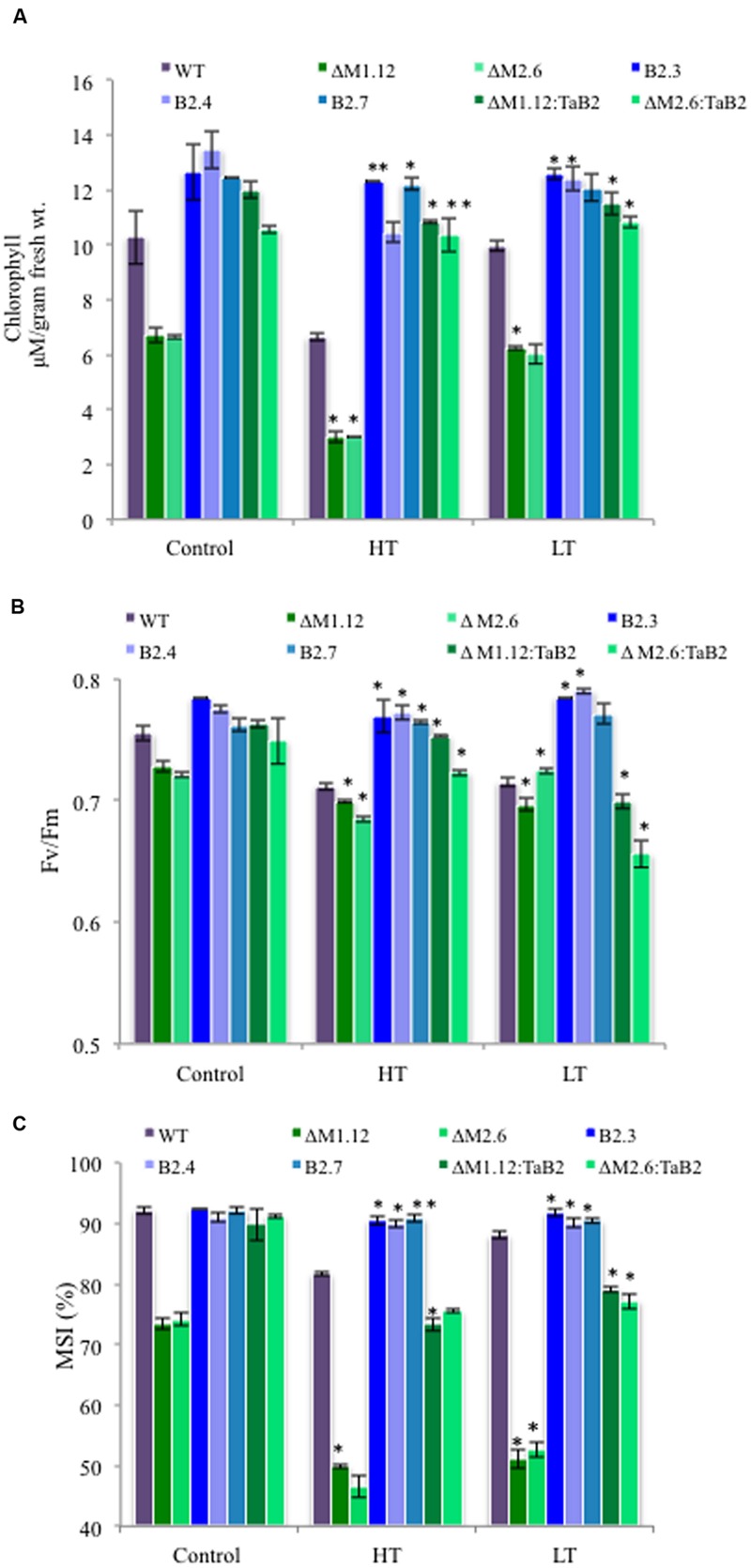
**Effect of extreme temperature stress on transgenic *Arabidopsis* plants. (A)** Chlorophyll content. **(B)** *F*v/*F*m. **(C)** Membrane stability index (MSI) of different *Arabidopsis* plants viz. WT, Δ*b2* (ΔM1.12 and ΔM2.6), *TaB2* -OE (B2.3. B2.4, and B2.7), and *C*Δ*b2* (ΔMI.I2:TaB2 and ΔM2.6:TaB2) lines under high temperature, HT (42°C) for 2 h and low temperature, LT (4°C) for 24 h treatment. Data represents from three independent replicates and are mean ± SD. Asterisks marked above bars indicate statistically significant differences (^∗^*P* ≤ 0.05, ^∗∗^*P* ≤ 0.01).

The tolerance to high and low temperature stress was further confirmed on the basis of physiological experiments of transgenic *Arabidopsis* plants analyzed quantitatively by measuring chlorophyll content, photosynthetic efficiency, and membrane stability index. The *TaB2*-OE transgenic and *C*Δ*b2* plants both under high and low temperature stress conditions showed higher chlorophyll content as compared to the WT plants. Interestingly, in Δ*b2* plants the extent of depletion in chlorophyll content was higher. Similar trend was also observed under non- stressed condition but the total chlorophyll content was relatively higher in all the different *Arabidopsis* plants as compared to the stressed condition (**Figure 9A**). The ability of transgenic plants to tolerate high temperatures was also checked by measuring the photosynthetic yield (*F*v/*F*m) of PSII which displayed higher value of *F*v/*F*m in overexpressing plants as compared to the WT and *C*Δ*b2* plants. The Δ*b2* plants exhibited reduction in PSII activity while minor differences observed between control and low temperature stress conditions (**Figure 9B**). Further, membrane stability index analysis was undertaken to assess stress tolerance which depicted that membranes of *TaB2*-OE plants showed higher stability under high and low temperature stress as compared to the WT and *C*Δ*b2* plants whereas Δ*b2* plants demonstrated drastic reduction in percentage of membrane stability index (**Figure 9C**). Thus, by monitoring the above physiological parameters, it is confirmed the ability of *TaB2*-OE transgenics to tolerate adverse temperature conditions efficiently.

### Lipid Peroxidation and *In Situ* Accumulation of Superoxide Radicals

**FIGURE 10:**
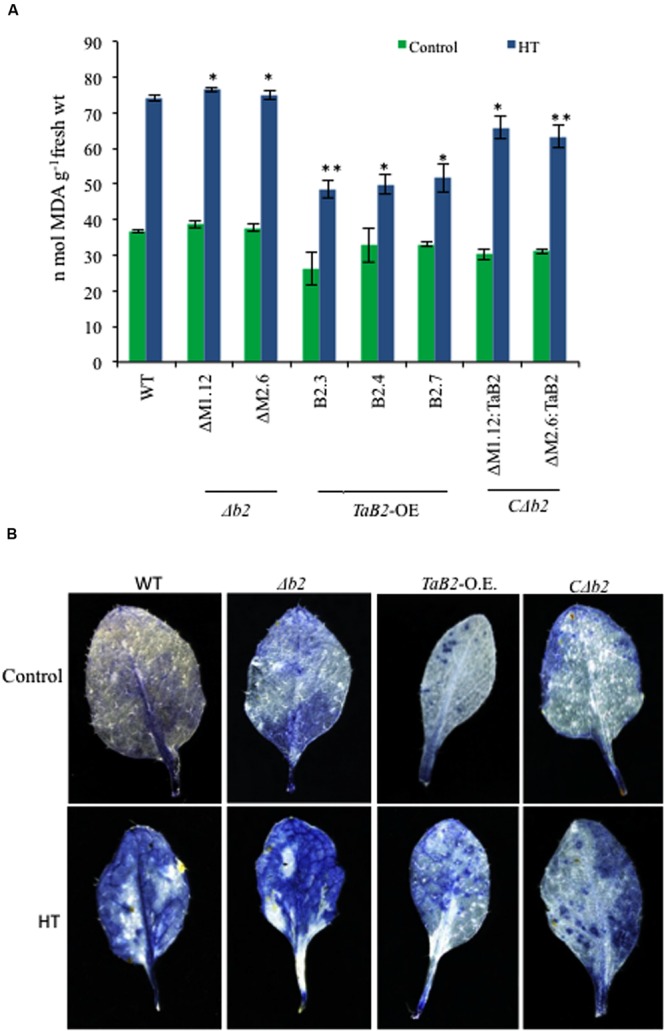
**Effect of HT stress on lipid peroxidation and superoxide levels in different *Arabidopsis* plants. (A)** Lipid peroxidation expressed as MDA content in seedlings of WT, Δ*b2, TaB2* -OE, and *C*Δ*b2* lines grown for 21 days on MS medium after heat stress given at 42°C for 2 h. **(B)** 2-week-old *Arabidopsis* leaf samples were stained for accumulation of $O_{2}^{•–}$ by NBT staining after 2 h of heat stress treatment at 40°C. Photographs were taken in stereo microscope Leica DFC 290 HD. Asterisks marked above bars indicate statistically significant differences (^∗^*P* ≤ 0.05, ^∗∗^*P* ≤ 0.01).

Lipid peroxidation was represented by estimating the level of malondialdehyde (MDA) level under heat stress conditions (Hameed et al., 2012). Heat stress significantly enhanced membrane deterioration as reflected by increased MDA content among all the *Arabidopsis* lines (**Figure 10A**). The MDA content between WT, Δ*b2*, transgenic and *C*Δ*b2* lines did not differ significantly under control temperature conditions, but when exposed to high temperature conditions the MDA levels in Δ*b2* plants increased, as compared to the *TaB2*-OE transgenic plants. Thus, the *Arabidopsis* plants transformed with *TaB2* showed better resistance to heat stress. To determine the effective role of reactive oxygen species (ROS) level in heat stress tolerance, we measured the superoxide ($O_{2}^{•–}$) levels. The leaves of WT and Δ*b2* plants showed higher accumulation of $O_{2}^{•–}$ under heat stress (**Figure 10B**) relative to that under control conditions. However, leaves of transgenic and *C*Δ*b2* plants partially stained with NBT which implies lower accumulation of $O_{2}^{•–}$ during high temperature stress conditions, suggesting that the tolerance level of transgenic lines was better under heat stress.

### Overexpression of *TaB2* in *Arabidopsis* Renders the Plant Hypersensitive to ABA

**FIGURE 11:**
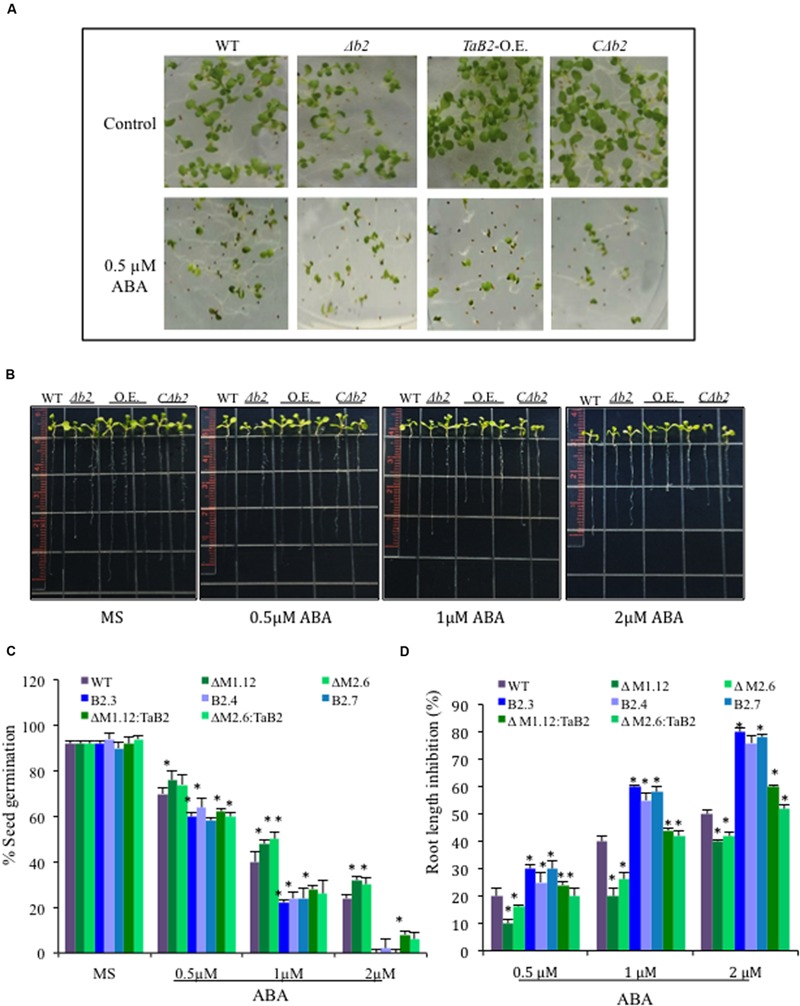
**Sensitivity of ABA on overexpression *TaB2* transgenic plant. (A)** Effect of different concentration of ABA on seed germination of *Arabidopsis* seedlings germinated on MS medium for 3 days. **(B)** Root growth inhibition phenotype of *TaB2-OE* transgenic lines. **(C)** Comparison of percentage of seed germination on different transgenic *Arabidopsis* seedlings treated with 0.5, 1, and 2 μM ABA. The number of germinated seeds was expressed as the percentage of total number (40–50) seeds plated. **(D)** Root length inhibition of WT, Δ*b2, TaB2* -OE, and *C*Δ*b2* seedlings treated with 0.5, 1, and 2 μM ABA supplemented MS medium for 4 days, calculated as the percentage of ABA treated seedlings relative to that on the ABA- free MS medium. Data represents from three independent replicates and arc mean ± SD. Asterisks above bars indicate statistically significant differences (^∗^*P* ≤ 0.05, ^∗∗^*P* ≤ 0.01).

To check the response of *TaB2 Arabidopsis* transgenic plants in presence of the stress hormone ABA, both germination and root growth inhibition assays were performed at different concentrations of ABA and compared amongst transgenic lines and WT along with the Δ*b2* and its *C*Δ*b2* lines. *TaB2*-OE seeds were found to be hypersensitive to ABA whereas seeds of Δ*b2* lines were more tolerant than the WT and *C*Δ*b2* lines even at higher concentration (2 μM) of ABA (**Figure 11A**). The difference in percent germination of overexpression lines was drastically reduced at higher concentration of ABA whereas, Δ*b2* seeds maintained the germination potential (**Figure 11C**). In addition, root growth inhibition assay was also performed and *Arabidopsis* seedlings were vertically grown on half-strength MS medium plates supplemented with 0.5, 1, and 2 μM ABA grown for 4 days and analyzed. The data presented in (**Figures 11B,D**) displays that *TaB2*-OE seedlings were oversensitive to ABA and with increasing concentration root growth inhibited drastically. The WT and *C*Δ*b2* lines maintained normal root growth at lower concentration of ABA while showed inhibition at higher concentration as compared to the Δ*b2* lines. These observations thus suggest hypersensitivity of *TaB2*-OE transgenic *Arabidopsis* plants toward stress hormone ABA.

## Discussion

In this study we describe the isolation and cloning of putative B2 protein from wheat ESTs obtained from the developing seed cDNA library and its functional characterization in *Arabidopsis* to investigate its role during plant development and abiotic stress. Originally, B2 protein was identified in suspension cultured carrot cells which was initiated during somatic embryogenesis (Schrader et al., 1997). Sequence analysis of B2 protein depicted a DCD domain in the carboxyl terminus which was predicted to be involved in development and cell death and found to be highly conserved in the plant species. Proteins harboring DCD domain categorized into four groups among which B2 protein is a member of group I of this family. *Arabidopsis* transcript profiling with microarray data elucidated its expression in embryo development and in shoot apex meristem organization (Tenhaken et al., 2005). Identification of other members of group I-proteins also revealed their function in either development (Li et al., 1998), abiotic stress response (Hoepflinger et al., 2011), phytohormone response (Li et al., 1998) and pathogen response (Ludwig and Tenhaken, 2001). Since, the exact function of B2 protein is unknown we were interested to define its role during plant development and in response to varied abiotic stresses. Therefore, we analyzed its genomic distribution, sub-cellular localization, expression profile during major developmental stages of wheat as well as under different abiotic stresses. Further to functionally characterize wheat B2 protein, we raised overexpressing transgenic *Arabidopsis* plants of *TaB2* and analyzed its role both during growth and development and in response to abiotic stress.

The deduced amino acid sequence contains the characteristic DCD domain of 132 residues at the C-terminal of polypeptide which takes part in plant development and cell death (Tenhaken et al., 2005). The putative TaB2 protein shares 100% sequence identity with the *Triticum urartu* GDA-1 protein (Dong et al., 2012) which is also one of the member of group-I family of DCD domain. B2 protein of *T. aestivum* upon multiple sequence alignment showed highly conserved amino acid residues at its C- terminal with respect to other plant species indicating a specific role of this consensus sequence in structure and function (Tenhaken et al., 2005). To decipher the evolutionary relationship of wheat B2 protein with other known as well as predicted B2 protein from different monocot and dicot plant species, a phylogenetic tree was constructed by multiple sequence alignment. The putative B2 protein from wheat shared 99% sequence similarity with *H. vulgare* B2 protein (not yet annotated), closely grouped in the unrooted tree and a similarity of 59% with *A. thaliana* putative B2 protein which was grouped in a different cluster with other dicot species. It also showed close relationship with other monocot species grouping one clade together with highest bootstrap values clearly demarcating between monocots and dicots, suggesting less specificity with dicot species during evolution. Nevertheless, due to its variable length and different architecture, DCD domain predicted to play an important role in protein–protein interaction (Tenhaken et al., 2005). The B2 protein was localized in nucleus and cytoplasm which was also predicted by *in silico* analysis by WoLF PSORT and NLS.

In the present study, we show differential expression of *TaB2* under different abiotic stresses and hormone treated vegetative (root and shoot) tissues and at major development stages of wheat specifically during heat stress. *TaB2* showed higher expression in root and shoots by approximately fuvefold change after 2 h of heat stress at 42°C followed by a moderately enhanced expression in NaCl, drought, 37°C and cold stress indicating the importance of *TaB2* during abiotic stresses particularly in high temperature stress. Expression of *TaB2* in various developmental stages of wheat concomitant with embryogenic stages were also examined where an increased level of expression was observed at 15 DAA followed by 20 DAA developing seed stage upon high temperature stress. Expression in heat stressed anther tissues, however, decreased followed by spike and ovary tissues. Summarizing the differential expression data, the role of TaB2 protein in plant development specifically in developing seed and in abiotic stress. Similar expression was also observed in one of the member of group-I protein family, *NRP* gene which showed higher expression during seed germination, flowers, and siliques predicting its importance in plant development and stress response (Hoepflinger et al., 2011). We also examined the transcript level of *TaB2* in presence of hormonal stimuli to investigate its possible role in plant development. Pronounced expression levels of *TaB2* were observed in vegetative tissues of wheat in response to ABA, which plays a crucial role in seed development (Finkelstein et al., 2002) and stress responses (Tuteja, 2007). Comparatively, low level of *TaB2* expression were demonstrated in presence of BR, another major plant hormone involved in plant growth and development, abiotic stress response and plant defense responses (Bari and Jones, 2009). Slightly enhanced expression in presence of SA predicted that *TaB2* might play a role in plant defense response similar to the other members of its group like *NRP* gene (Ludwig and Tenhaken, 2001).

Since, very limited information actuated on the function of this plant protein, we studied its involvement in plant development by examining morphometric characteristics. For this, we generated overexpression transgenic lines of *TaB2* in *Arabidopsis*, identified T-DNA insertion Δ*b2* mutants and developed its complemented lines to examine the phenotypic differences. The *TaB2* overexpression *Arabidopsis* plants exhibited enhanced plant growth, larger rosette diameter, increased rosette leaf number, leaf length, and leaf width, higher silique number and longer silique length as compared to the wild-type and complemented plants which is significantly similar to each other whereas, mutant plants characterized by stunted plant growth, decrease in rosette diameter and rosette leaf number, shorter leaf and reduced number of siliques and silique length, respectively. This drastic difference in phenotype of Δ*b2* mutant confirms the importance of this protein in growth and development.

The differential expression levels of *TaB2* observed in vegetative and reproductive wheat tissues led us to investigate the tissue-specific expression of transgenic *TaB2* in *Arabidopsis* plants to analyze its transcript level under control and heat stressed conditions. Since, flowers were more sensitive toward high temperature and seeds were heat stress tolerant, the expression of *TaB2* was found to be up-regulated in developing and mature siliques under heat stressed condition whereas the expression decreases in leaf and flower tissues thus assuming its function in developing seed stages, embryogenesis, and high temperature stress conditions.

Wheat is one of the major food crop which is prone to extreme temperature stress declining its productivity worldwide. High temperature stress at the grain filling stage critically affects the yield (Abrol and Ingram, 1996). Heat and cold stress both causes severe damage to the plants affecting the morphological, physiological, and molecular changes where the variety of genes adapt to tolerate these stresses. In order to explore functions of TaB2 protein in stress tolerance, overexpression transgenic *Arabidopsis* lines were raised and their phenotype analyzed. Phenotypic difference in hypocotyl elongation observed between WT, *TaB2*-OE, Δ*b2* and its *C*Δ*b2 Arabidopsis* seedlings. Under control conditions, elongated hypocotyl monitored in *TaB2*-OE seedlings represent shorter hypocotyl, while the *C*Δ*b2* seedlings regain the elongated phenotype. Also, we checked the hypocotyl elongation under heat stressed condition where a similar trend was observed but with slight reduction in hypocotyl length as compared to the control conditions. The results obtained indicate that the elongation might be due to*TaB2* gene action and depicts its role in plant development as well as tolerance to high temperature stress response. The percentage increase in root length and increase in other parameters like plant height and rosette diameter of *TaB2*-OEplants in presence of high and low temperature stress in comparison to the Δ*b2* plants implies enhanced tolerance and development of plants under control conditions. Hypocotyl and root growth inhibition of Δ*b2* seedlings under stress conditions inferres importance of B2 protein for development and stress adaptations likewise to its other proteins (NRP, Gda-I) of this group-I family of DCD domain (Li et al., 1998; Hoepflinger et al., 2011). Since heat is known to damage several parts of the cell, affecting most cellular processes (Munro and Pelham, 1985; Larkindale and Knight, 2002), we analyzed physiological parameters like chlorophyll content, photosynthetic yield and membrane stability index to examine the effect of stress. Chlorophyll fluorescence parameter *F*v/*F*m which represents maximum quantum yield of PSII (Ibaraki and Murakami, 2007), chlorophyll content and membrane stability index which are indicators of membrane damage (Dhindsa et al., 1981; Sairam et al., 1997) were affected by adverse temperature stress. *TaB2* transgenic plants exhibited higher chlorophyll content, photosynthetic yield (*F*v/*F*m) and better membrane stability index under temperature stress conditions and reflects the role of B2 protein in heat and cold stress responses. Heat stress causes enhanced production of ROS in cells that manifest as lipid peroxidation by the disruption of cellular homeostasis and the uncoupling of metabolic processes (Larkindale and Knight, 2002; Suzuki and Mittler, 2006). ROS are capable of controlling expression of various genes, hence controlling different processes like programmed cell death (PCD), development and growth, pathogen defense, abiotic stress responses, and others (Gill and Tuteja, 2010). Interestingly, in our study we observed lower level of lipid peroxidation and reduced accumulation of $O_{2}^{•–}$ in *TaB2*-OE plants under high temperature stress conditions which indicates that TaB2 protein may play a protective role during stress tolerance also.

The phytohormone ABA mediates various facets of developmental processes such as seed maturation, germination, dormancy, and in abiotic stress adaptations (Leung and Giraudat, 1998; Yoshida et al., 2006). Since, expression of *TaB2* is upregulated in developing seeds of wheat and overexpression lines of *Arabidopsis* as well as showed tolerance at adverse temperature stress we were interested to study the response of *TaB2-*OE lines in presence of ABA. The results obtained from the phenotype analyses indicates that the *TaB2*-OE seedlings are hypersensitive to ABA in both the seed germination assay and root growth elongation assay whereas Δ*b2* seedlings performed better than the OE, WT and *C*Δ*b2* lines in response to ABA. Similarly, ABF3 or ABF4 overexpression lines which is a ABA-responsive element binding factor were also found to be ABA hypersensitive at both the germination and seedling growth stages (Kang et al., 2002) therefore, it can be assumed that *TaB2-*OE may be modulating development or retardating growth probably by the constitutive operation of part of the ABA signal transduction cascade.

## Conclusion

**FIGURE 12:**
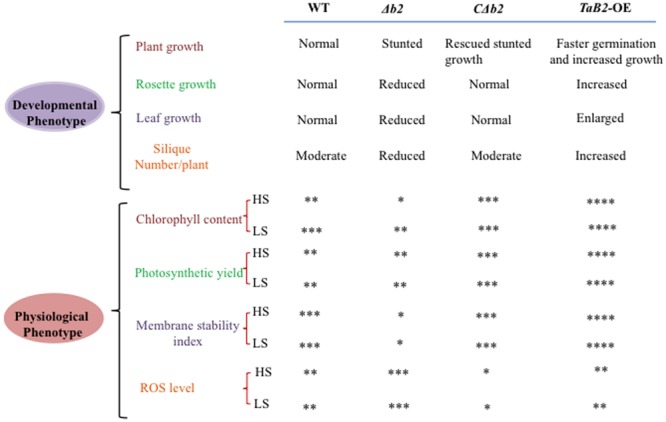
**Comparative evaluation of the effects of WT, Δ*b2, C*Δ*b2*, and *TaB2-OE* on developmental and physiological phenotypes.** Level of physiological parameters represent by ^∗^ sign. Very low (^∗^), low (^∗∗^), high (^∗∗∗^), very high (^∗∗∗∗^) at high temperature stress (HS) and low temperature stress (LS), respectively.

It is evident from the above study that *TaB2* expressed in developing seeds of wheat and also in developing and mature siliques of *Arabidopsis* is developmentally linked to abiotic stress tolerance. Moreover, *TaB2*-OE lines in *Arabidopsis* showed overall enhanced plant growth and its T- DNA insertion Δ*b2* line representing stunted growth indicates the importance of this gene in plant growth and development (**Figure 12**). Interestingly, overexpression lines exhibits elongated hypocotyl, better root growth, increased level of chlorophyll, better adaptation in photosynthetic yield, membrane stability, and lower accumulation of superoxide ion compared to the WT under high as well as low temperature stress deciphering the functional role of TaB2 protein in stress response and signaling. ABA hypersensitivity of transgenic plants suggests a possible path of ABA-dependent growth regulation of seedlings. It will be of interest to explore the complexity of ABA mechanism in the functioning of B2 protein and further analysis in response to stress adaptation needs to be further elucidated.

## Author Contributions

AS conducted the experiments and wrote the manuscript draft. PK conceived the idea and implemented the experimentation, discussed the results, and finalized the ms.

## Conflict of Interest Statement

The authors declare that the research was conducted in the absence of any commercial or financial relationships that could be construed as a potential conflict of interest.

## Footnotes

1 http://www.generunner.com

2 http://www.genenames.com

3 http://smart.embl-heidelberg.de/

4 http://www.ncbi.nlm.nih.gov

5 http://signal.salk.edu/isects.html
