# Supplementary material for: Molecular and Functional Characterization of a Wheat B2 Protein Imparting Adverse Temperature Tolerance and Influencing Plant Growth
Source: Front Plant Sci. 2016 May 10;7:642. doi: 10.3389/fpls.2016.00642 (PMC4861841; doi:10.3389/fpls.2016.00642)
Supplement: Supplementary file 1 [file Data_Sheet_1.PDF]

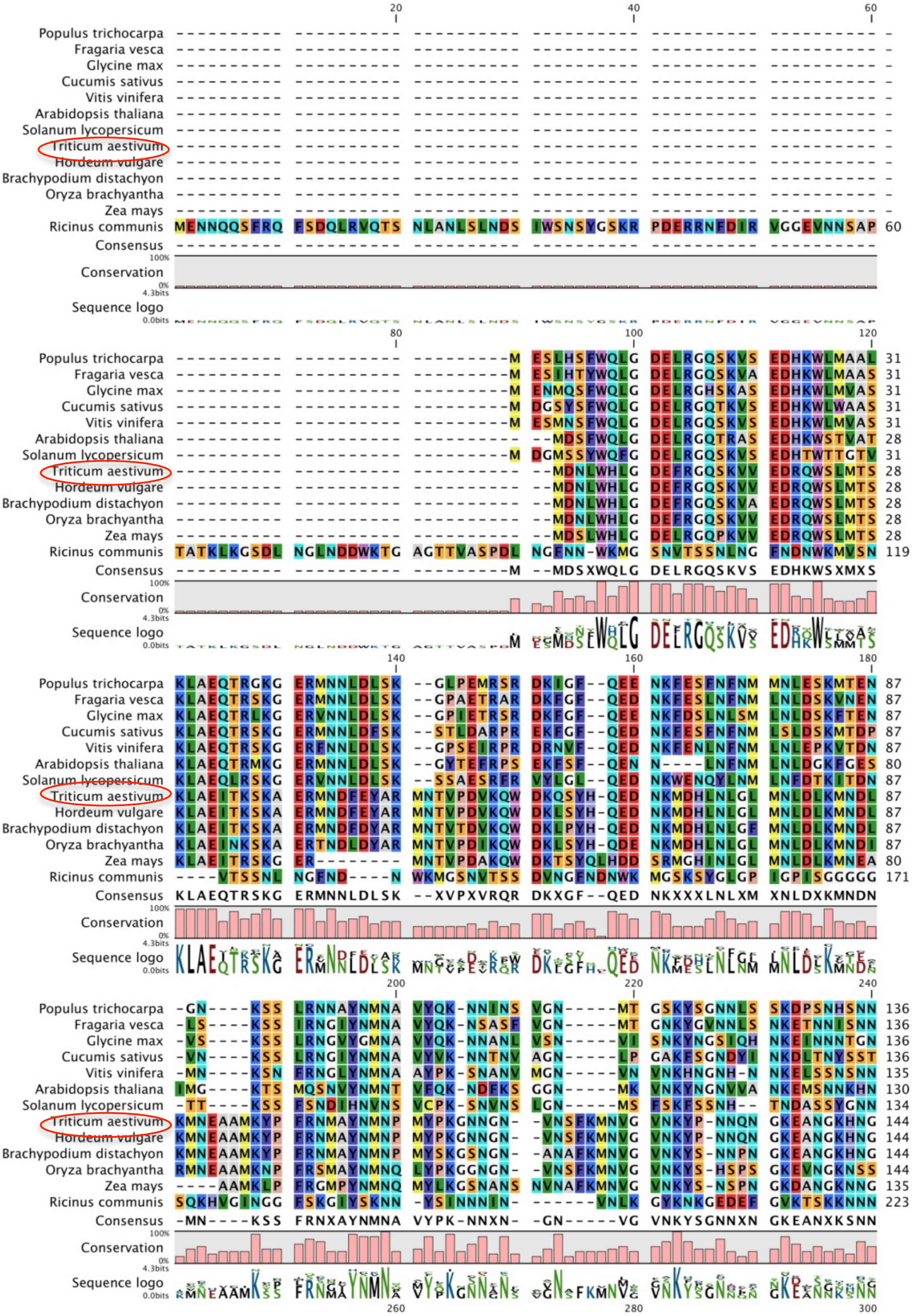
Populus trichocarpa
Fragaria vesca
Glycine max
Cucumis sativus
Vitis vinifera
Arabidopsis thaliana
Solanum lycopersicum
Triticum aestivum
Hordeum vulgare
Brachypodium distachyon
Oryza brachyantha
Zea mays
Ricinus communis
Consensus
Conservation
Sequence logo
100%
0%
4.3bits
0.0bits

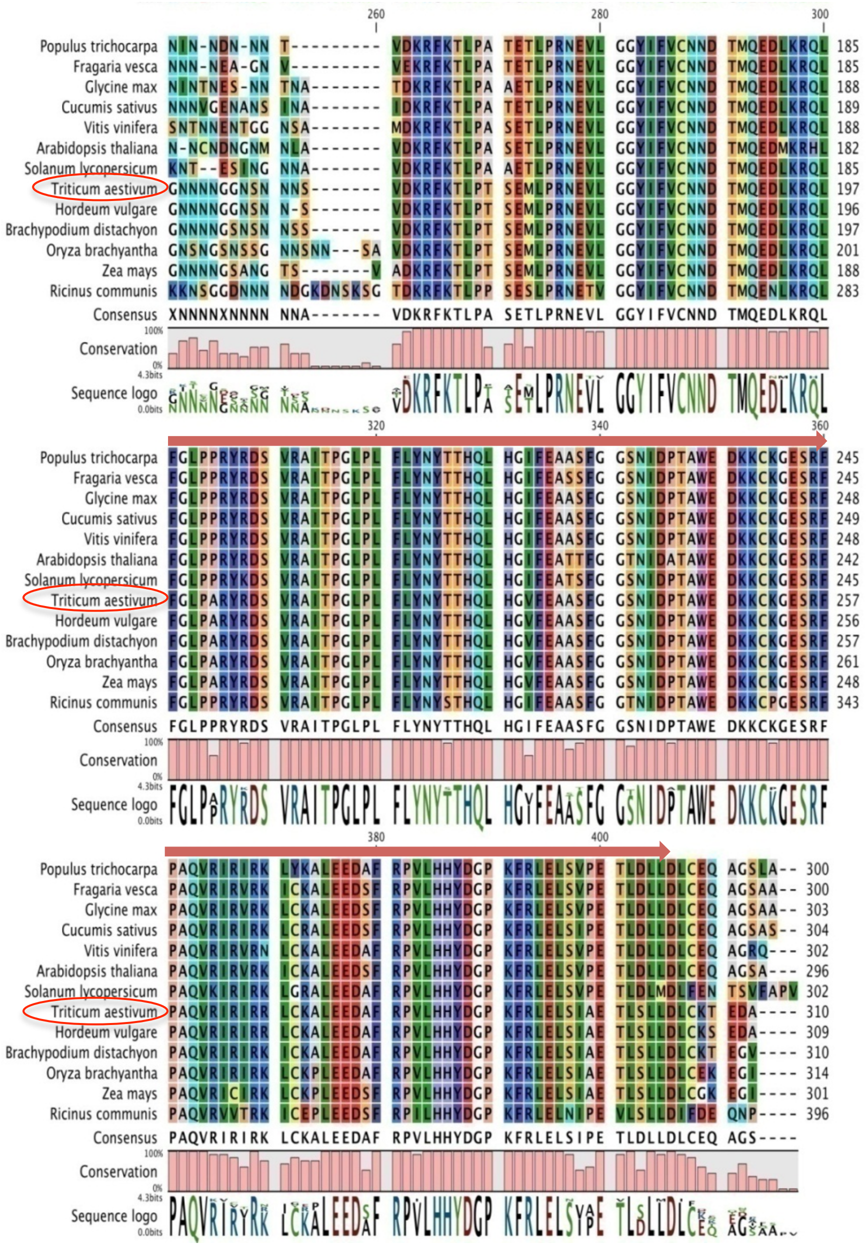

Supplementary Figure S1 Multiple sequence alignment of *T. aestivum* putative B2 proteins showing homology and conserved amino acid residues with other plant organisms having characteristic plant domain, development and cell death (DCD) at its carboxyl terminus represented by red arrow respectively.

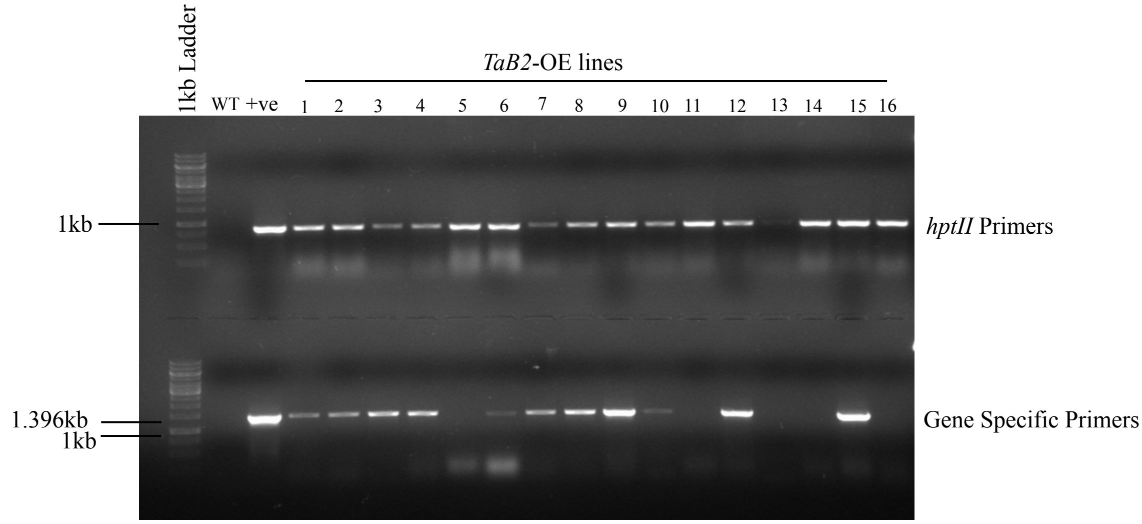

Supplementary Figure S2 Confirmation of putative transformants of *TaB2* in *Arabidopsis* transgenics by RT-PCR. First strand cDNA constructed by Superscript III first strand cDNA synthesis system (Life Technologies, USA) using RNA from leaf samples of WT and different transgenics of *TaB2 Arabidopsis* lines and confirmed through PCR with *hptII* and *TaB2* specific primers.

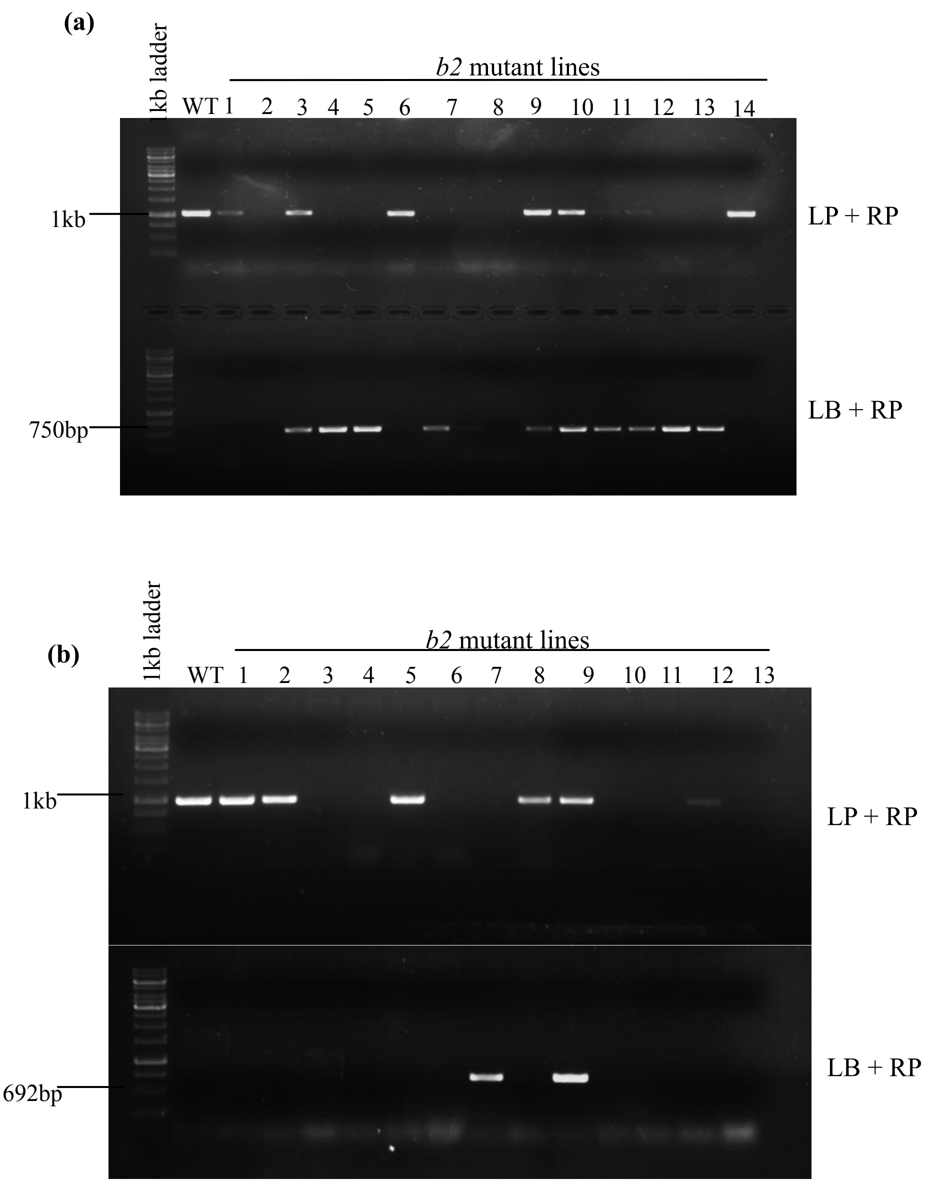

Supplementary Figure S3. Screening of *Arabidopsis b2* mutant SALK_044868 and SALK_041306 lines for homozygous plants. Two paired reactions of *b2* mutant lines (A) SALK_044868 and (B) SALK_041306 using left border primer of T-DNA insertion (LB) with right gene-specific primer (RP) in one reaction and left gene specific (LP) with right gene specfic primer (RP).

Supplementary Table S1 Sequence of primers used in the experiment

Gene	Primer ID	Sequence (5'-3')
TaB2	5'RACE (GSP1)	GAGGTAGACCAGGAGTGAT
	N5'RACE (NGSP1)	CCACCAAGAACTTCATTCCTCGGTAGC
	3'RACE (GSP2)	CGCCTGGAGCTCTCCATAGCAGAGACACTG
	N3'RACE (NGSP2)	CGGAACATGGTTGTGGTTGCTCTGTG
	Topo-*TaB2*-F	CACCATGGACAACTTGTGGCATCTCGG
	Topo-*TaB2*-R	TCAGGCGTCTTCTGTCTTGCACAGGTC
	Fwd (Real Time)	GCCAGCAAGATATCGTGATTCA
	Rev (Real Time)	TGGGTCGTGTAGTTGTAGAGGAAA
Ta-Actin	Forward	TTGCACCAAGCAGCATGAA
	Reverse	AACCACCGATCCAGACACTGTA
At-Actin	Forward	ATCAGCCGTTTTGAATCTCCGG
	Reverse	GCCTTTGGGTTAAGAGGAGCCTC
hptII	Forward	TCTACACAGCCATCGGTCCAG
	Reverse	GATGTAGGAGGGCGTGGATAT
SALK_044868 (ΔM1.12)	Left genomic primer (LP)	GTGACACAACCAACAAAGCTG
	Right genomic primer (RP)	ATTGTAATGCTGCAAACCAGG
SALK_041306 (ΔM2.6)	Left genomic primer (LP)	TTCTCCCTATTTTTGGGGTTG
	Right genomic primer (RP)	CAGAGGAACGAAGACGACAAG
